# An uneven playing field: a mixed methods, multiphase feasibility study of a programme to reduce gambling among at-risk men in a professional football club setting

**DOI:** 10.1186/s12889-026-26845-z

**Published:** 2026-03-05

**Authors:** Blair Biggar, Christopher Bunn, Gerda Reith, Heather Wardle, Craig Donnachie, Manuela Deidda, Frankie Graham, Cindy Gray, Nicola Greenlaw, Kate Hunt, Matthew Philpott, Neil Platt, Robert Rogers, John Rooksby, Sally Wyke

**Affiliations:** 1 Sociology, School of Social and Political Sciences, University of Glasgow, Glasgow, Scotland; 2 Department of Psychological Sciences and Health, University of Strathclyde, Glasgow, Scotland; 3 Computer and Information Sciences, Northumbria University, Newcastle upon Tyne, England; 4 Health Economics and Health Technology Assessment, School of Health and Wellbeing, University of Glasgow, Glasgow, Scotland; 5 Healthy Stadia, Liverpool, England; 6 Bet Know More, London, England; 7 Beacon Counselling Trust, Liverpool, England; 8 Robertson Centre for Biostatistics, School of Health and Wellbeing, University of Glasgow, Glasgow, Scotland; 9 Institute for Social Marketing, University of Stirling, Stirling, Scotland; 10 School of Psychology and Sport Science, Bangor University, Bangor, Wales

**Keywords:** Gambling, Betting, Football, Public health, Intervention, Gambling behaviour change

## Abstract

**Background:**

Sports betting is a growth area for the gambling industry, with football fans a key target of advertising. Men are particularly at risk from gambling harm. The 8-week Football Fans and Betting (FFAB) intervention was designed for delivery in professional football clubs by club community coaches to reduce sports betting and other forms of gambling among men aged 18–55 with a PGSI score of < 15. This paper reports the acceptability and feasibility of delivering FFAB in England.

**Methods:**

We conducted a multiphase, mixed methods, feasibility study of the FFAB intervention, assessing feasibility through three criteria: recruitment, fidelity and acceptability. We generated quantitative process and attendance data to assess recruitment and retention, and observation, interview and focus group data to examine fidelity and acceptability. Quantitative data were analysed descriptively, qualitative data using thematic analysis, before being combined using triangulation protocol to assess feasibility against the three criteria.

**Results:**

FFAB was launched in six clubs. Despite multiple attempts to refine and improve recruitment processes, no club was able to recruit the target number of participants to the programme. Retention on the programme also faced challenges. The saturation of football’s commercial landscape by the gambling industry and stigma-related social dynamics appear to underpin these findings. Due to recruitment and retention challenges, fidelity criteria were not met. However, participants that attended the programme, and coaches that delivered it, reported that FFAB was acceptable and supported some to make changes to their gambling behaviours. Throughout the qualitative dataset, participants and coaches emphasised the necessity of intervention to prevent gambling harms among men.

**Conclusions:**

We found that FFAB was acceptable to the coaches who delivered it and the participants who attended. However, our model for recruitment did not work, with consequences for fidelity. We also faced difficulties with retention. More feasibility work to develop a different approach to a gambling reduction with men between 18 and 55 with a PGSI score of less than 15 is required.

**Supplementary Information:**

The online version contains supplementary material available at 10.1186/s12889-026-26845-z.

## Background

Gambling negatively impacts the health and wellbeing of individuals, families, communities, and society [1, 2]. Public health approaches conceptualise these impacts as ‘gambling harms’. Gambling harms include financial and relationship problems, stress, depression, and anxiety [3, 4], which, for some people, leads to self-harm, suicidal ideation, and suicide attempts [5, 6]. Such harms frequently extend beyond people who gamble to affect their families, friends and wider communities. In the UK, the cost associated with harmful gambling is conservatively estimated to be between £1.05 and £1.77 billion [7]. Leading evidence suggests that mitigating gambling harms requires concerted legislative action to restrict the industry [2]. Alongside this essential action, selective and targeted public health interventions have an important role to play, with a range of approaches demonstrating efficacy [2].

In the UK, sports betting is a substantial sector within the gambling industry, reaching a gross yield in excess of £5bn in 2023-24, about a third of the overall Gross Gambling Yield of £15.6bn in the same period [8]. Gambling companies are investing heavily in the sponsorship of English Football clubs [9, 10], advertising in stadia [11], via broadcasts [12], and through a range of online channels [13], frequently promoting in-play betting to fans [14]. These investments target a key demographic – the male sports fan – in social spaces they frequent and dominate. Approximately one in six men engaged in sports betting in the past year in the UK [11], yet less than 10% of those experiencing gambling harms seek support [15, 16]. Recent data from the Gambling Survey for Great Britain reinforce these findings, indicating that only around 1% of those who have gambled in the last year sought help from gambling support services, while about 3% reported experiencing at least once severe consequence [17]. In the same survey, men consistently experienced severe consequences from their own gambling at a higher rate than women (3.5% vs. 2.1%).

The stigma associated with the experience of gambling harms is a key factor in low rates of support-seeking, especially among those who hide or have been ostracised due to their gambling [18]. In other areas of public health, collaboration with professional sports organisations has been pursued to reduce stigmatisation of health problems and encourage help seeking. A leading example of this approach is the Football Fans in Training (FFIT) programme, which collaborated with professional football clubs [19] to de-stigmatise weight management among men. FFIT is a 12-week, gender-sensitised, group-based, weight management and healthy living programme [20] which succeeds in using the appeal of professional football clubs [21] to recruit men and support them to lose weight [19]. Subsequent research demonstrated that weight loss and other positive behavioural changes were sustained in the longer term [22]. FFIT was initially developed, evaluated, and implemented in Scotland before its success led to the programme being scaled ‘up’ and ‘out’ to new sports, settings, target behaviours, and populations [23, 24].

Inspired by this model, we set out to examine whether collaboration with professional football clubs could engage men at risk of experiencing severe gambling harms, by conferring legitimacy on an otherwise stigmatised activity- help seeking. We developed the Football Fans and Betting (FFAB) programme to support men aged 18–55 who regularly bet on sports to reduce their gambling, in an attempt to address the absence of gender-sensitive approaches to gambling harm prevention among men [25]. In the study we report on here, we aimed to assess whether FFAB could become a feasible addition to the range of existing, under-utilised, treatment and support options available in Britain e.g. the National Gambling Helpline, peer support groups, online support, counselling and therapy services. Feasibility was assessed against a range of pre-defined criteria including: recruitment rates, acceptability of the programme and intervention fidelity (see Table 1).

**Table 1:** FFAB feasibility study progression criteria

Evaluation Domain	Progression Criteria
1. Recruitment	a. Recruitment of at least 60% of participants across the four clubs within the two months scheduled for recruitment (progression criteria met) b. Recruitment of between 50% and 60% of participants across the four clubs within the two months scheduled for recruitment (propose rescue plan) c. Recruitment of < 50% of participants across the four clubs within the two months scheduled for recruitment (stop)
2. Fidelity	a. Research team agree, based on observations of sessions, that FFAB was delivered with reasonable fidelity and limited unintended consequences (progression criteria met) b. Research team agree, based on observations of sessions, that FFAB can be delivered with reasonable fidelity and limited unintended consequences (propose changes) c. Research team agree, based on observations of sessions, that FFAB cannot be delivered with reasonable fidelity and limited unintended consequences (stop).
3. Acceptability	a. Participants report that FFAB was useful, appropriate and acceptable (progression criteria met) b. Participants report that FFAB, with refinement, may be useful, appropriate and acceptable (propose changes) c. Majority of participants report that FFAB was not at all useful, appropriate or acceptable (stop). d. Coaches report that FFAB was appropriate and acceptable (progression criteria met) e. Coaches report that FFAB, with refinement, was appropriate and acceptable (propose change) f. Most coaches report that FFAB was not at all appropriate and acceptable (stop)

## Methods

### The FFAB Programme

FFAB is the first gambling harm prevention intervention of its kind to support men to reduce their gambling. FFAB drew inspiration from the model successfully utilised in the FFIT intervention. The underpinning conceptual framework of the FFAB intervention combines a range of techniques drawn from the Gambling Intervention System of Characterisation version 1 (GIST-1) and the Behaviour Change Taxonomy (BCT) [26, 27]. A detailed description of the FFAB intervention, following the TIDieR standard, can be found in Table 2 [28].

**Table 2:** TIDieR table for the football fans and betting programme

Name (1)	FFAB
Why (2)	Men who bet on sports are among the most at risk of experiencing gambling harms and are targeted by the gambling industry. The health harms from gambling can be severe, including self-harm, suicidal ideation and suicide attempts. Harms not only impact gamblers, but also their families, social networks, communities, and society. Very few seek formal treatment. The aim of FFAB is to help men aged 18–55 who bet on sports regularly (at least weekly) to reduce their gambling. FFAB draws on the Football Fans in Training and European Fans in Training models [19, 24] and is designed to attract men by appealing to interest in their football club and in being active/playing football with other men.
What, materials (3)	FFAB is grounded in self-determination theory (promoting autonomy, relatedness and competence in managing betting behaviours) [29], behaviour change techniques previously used in other gambling reduction interventions [26, 27], and sociological understandings of social networks, masculinity and gambling [30–32]. FFAB requires access to club facilities for weekly group discussion and physical activity sessions. Coach delivery manuals include the rationale, content and suggestions of how to deliver each weekly session. Coaches also receive a PowerPoint slide deck to support deliveries. Participants receive: a manual including information and self- monitoring forms; and access to a bespoke app called ‘Reclaim the Game’ that enables participants to track and set goals around their gambling practices.
What, procedure (4)	**Football clubs**: Football clubs opt in to delivering the FFAB programme. **Training**: Club community coaches are trained to deliver the programme over two days. Training is experiential and interactive and focuses on the ethos of the FFAB programme. It covers: the nature and extent of gambling harms; the risks men face and how to safeguard; promoting the use of self-monitoring, goal setting, problem-solving and action-planning; supporting men’s motivation to sustain behaviour changes; facilitating group discussions for experiential learning; fostering a safe and comfortable environment which enables men to support one another and develop autonomy. **Recruitment**: Clubs attract participants using various methods, including online promotion (such as advertisements on club or fan websites), email, newsletters, social media announcements (like Twitter and Facebook), posters, flyers, advertising during matches, direct recruitment at home games (distributing leaflets and gathering contact information), coverage in local or national media, engagement with local supporters’ groups, and recommendations through informal communication. **Content**: FFAB teaches tools to commence behaviour change followed by those to maintain it, framed by information that gives a critical insight into the tactics used by the gambling industry to attract and retain customers. Each session promotes peer interaction, experience-sharing, and enjoyment. Men are actively taught how to utilize a collection of specific behaviour change techniques, referred to as a “toolbox,” and are encouraged to experiment with and adopt those methods that prove effective and sustainable for them. This toolbox encompasses personalized and accessible health and lifestyle information, setting both behavioural and outcome goals, problem-solving, action planning, self-monitoring using a gambling diary (either through the ‘Reclaim the Game’ app or on paper), and establishing social support networks within the group (including through social media). The coaches assist men in adjusting behaviours that may challenge their masculine identities while still aligning with them, such as by focusing on acquiring new skills supported by evidence for managing lifestyle. The sessions additionally incorporate physical activity sessions at club venues to enhance social cohesion and encourage bonding and camaraderie (in addition to providing wider mental health/health benefits), where coaches motivate each participant to exercise at a level suitable for their own fitness and capabilities. As the programme progresses, men are supported in sustaining changes by incorporating the behaviour change methods they find beneficial and meaningful into their everyday routines (such as goal-setting, self-monitoring, and action planning), and by utilizing relapse prevention strategies. Coaches foster shared learning of maintenance strategies through group interaction and encourage a growing sense of connection to the club, the coach, and fellow participants. The significance of the changes already implemented is emphasized, with men being urged to discover methods of incorporating new behaviours into their daily schedules in a way that is both enjoyable and manageable. They are also prompted to acknowledge the advantages of behaviour change that are relevant and important to them (e.g. improved finances, better relationships, better mood).
Who provides (5); How (6); Where (7); How much (8)	Community coaches from professional football clubs conduct 8 weekly in-person sessions lasting 90 min each for groups of up to 15 men. These sessions take place at the club’s stadiums or training facilities, aiming to cultivate a sense of belonging by providing participants with an inside look at the club and increasing their physical and symbolic closeness to it. This, in turn, fosters a stronger connection to the club.

Numbers in parentheses refer to the item number on the TIDieR checklist [28]

TIDieR, Template for Intervention Description and Replication

The FFAB intervention is delivered by coaches working within club community trusts at professional football clubs in England over the course of an 8-week programme. Each of the eight sessions included 45 min of classroom-based content (for example learning about how the gambling industry generates profits, the scale and nature of gambling harms, and the tools and support opportunities available to anyone experiencing these harms) followed by 45 min of physical activity (for example, football-based skills and 5 aside games). A session-by-session overview of the FFAB programme’s aims, and the intervention techniques used to pursue them, is provided in Additional File 1.

### Feasibility study design

The FFAB feasibility study was intended to be the first stage in a sequence of studies, which progressed to a pilot randomised controlled trial (RCT) and then to full RCT. Progression criteria were developed to establish unambiguous requirements that had to be met at each stage of this research plan to enable progression to the next. The progression criteria for the feasibility phase are described in Table 1.

**Fig. 1:**
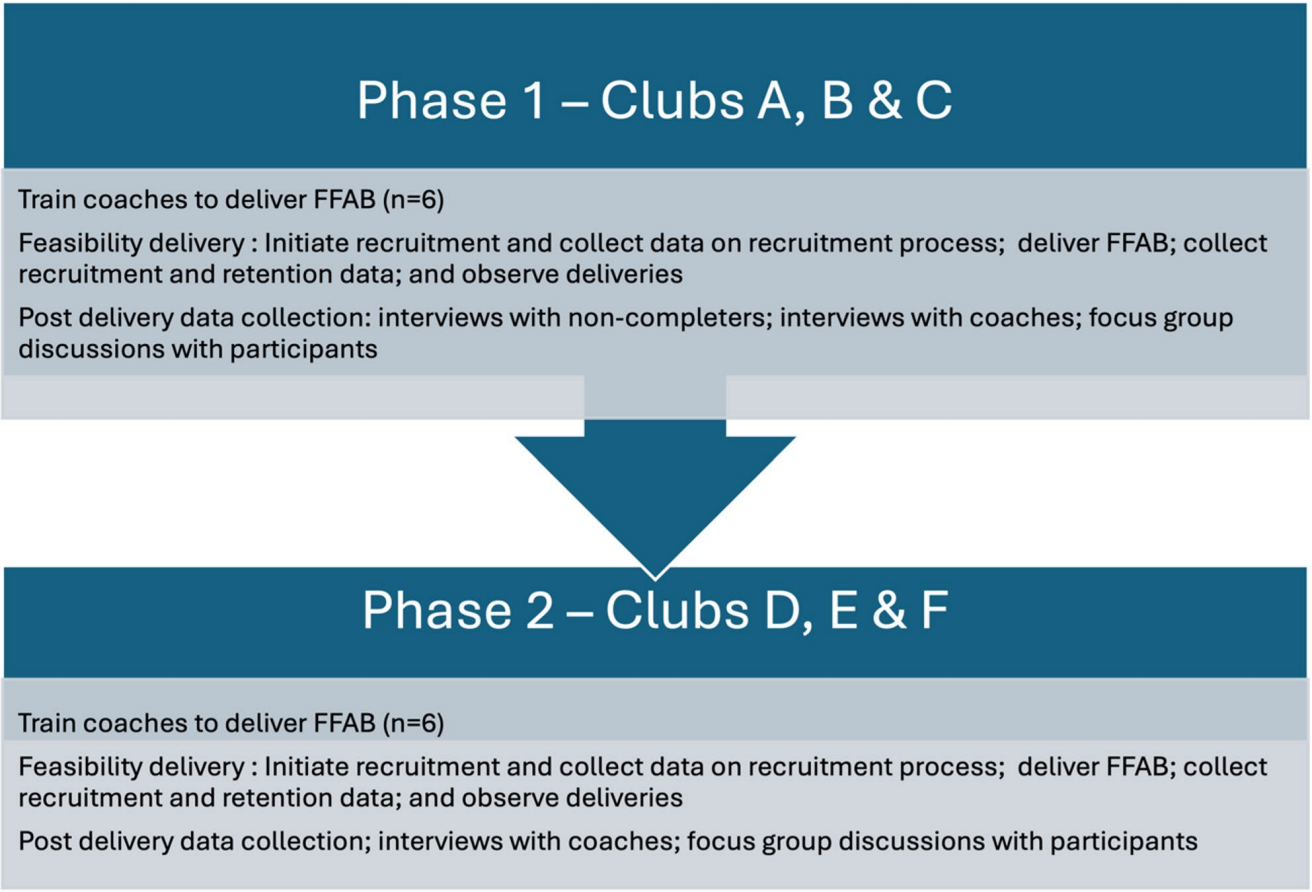
FFAB multiphase feasibility study design

To assess the feasibility of FFAB against the progression criteria, we adopted a multiphase, mixed methods, feasibility study design. This design was delivered in two phases, in partnership with six professional football clubs in England, as shown in Fig. 1. The two phases were intended to enable us to test and refine the FFAB programme using findings from the first phase to inform the second phase.

The FFAB project initially launched in January 2020 but was paused in March 2020 due to the onset of the Covid-19 pandemic. A gradual restart began in December 2021, with full resumption of activities by January 2022. This extended interruption significantly delayed both recruitment and programme delivery timelines.

### Recruiting clubs

Recruitment of clubs was facilitated through Healthy Stadia, a public health charity and project partner with established relationships across UK-based football clubs. Community trusts linked to football clubs were contacted by Healthy Stadia and invited to take part. Three clubs (Clubs A, B, and C) were recruited for Phase 1, and three additional clubs (Clubs D, E, and F) joined in Phase 2. Although none of the participating clubs had a gambling company as a shirt sponsor at the time of recruitment, two had existing commercial partnerships with gambling operators. Regardless of their individual sponsorship arrangements, all clubs outside of the Premier League were required to display SkyBet advertising due to league-wide sponsorship agreements.

### Participant recruitment and eligibility

Recruitment to FFAB was led by clubs and their community trusts, supported by the research team, with the aim of recruiting 10–15 eligible men per club. Clubs and their community trusts utilised a range of channels to target fans and other men in their local communities, including social media, Trust websites, flyers, posters, and matchday stadium activities (see Table 3), supported by a dedicated recruitment manual, provided by the researchers, containing exemplar materials. Adverts directed interested individuals to complete an online expression-of-interest form.

**Table 3:**
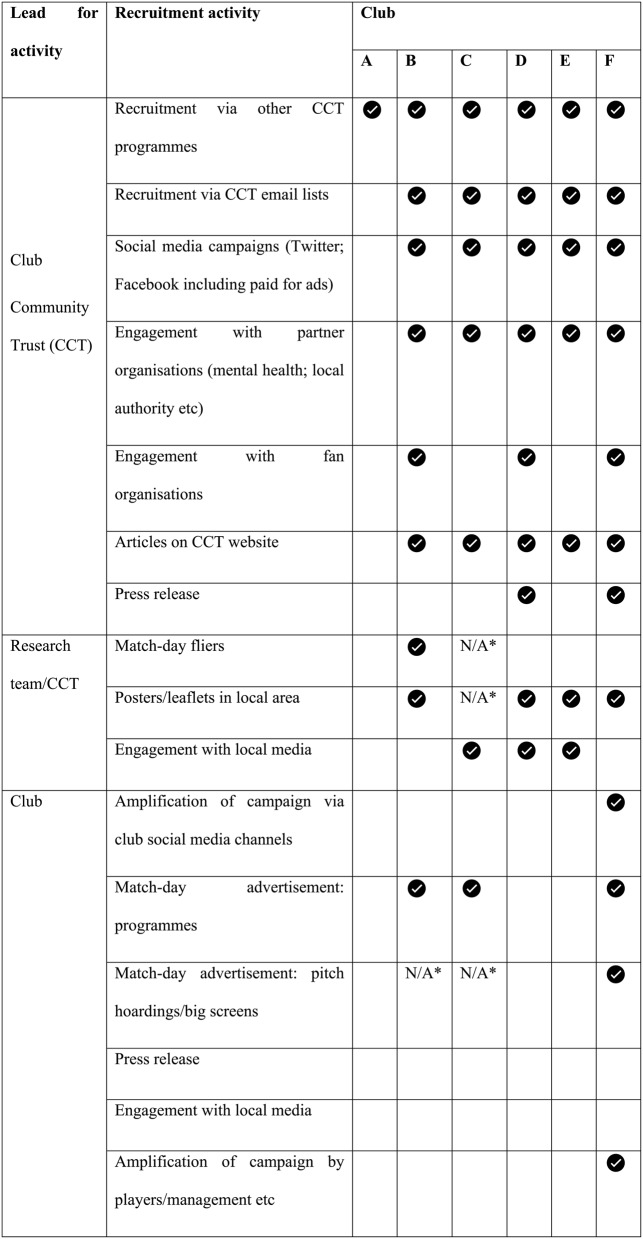
Recruitment activity by feasibility club (Phase 1 and 2)

Clubs A-C were part of Phase 1, while Clubs D-F were part of Phase 2

*Recruitment was out of season

Those who completed the form were contacted by researchers for screening against the inclusion criteria, using the Problem Gambling Severity Index (PGSI) to assess potential participants’ suitability for the programme. The PGSI is a widely used tool to gauge levels of gambling-related harm [4]. We used the long-form version, comprising nine questions about gambling behaviour over the past 12 months (e.g. betting more than one could afford to lose, or feeling guilty about gambling). Responses are rated on a four-point scale from “never” (0 points) to “almost always” (3 points), with scores categorised as follows: 1–2: low-risk, 3–7: moderate-risk, 8+: problem gambling.

In Phase 1 (see Fig. 1), participants were eligible if they were: (a) male, (b) aged 18–44, (c) gambled regularly (once a week or more), (d) expressed a desire to reduce their gambling, and (e) had a Problem Gambling Severity Index (PGSI) score of 8 or less. During Phase 2, the criteria were revised following consultation with the funder and discussions with clinical advisors from Beacon Counselling Trust. The PGSI cut-off was raised to 14, and the age range expanded to include men aged 18–55. We discuss this shift in inclusion criteria in detail below, in our findings section.

### Data collection

We collected a range of qualitative and quantitative data to enable us to assess the feasibility of the FFAB programme. Quantitative data captured included: the number of expressions of interest received from men at each club; the number who were eligible; and the number who joined and attended the programme. These data were collected via an online form (expression of interest) and via registers taken at each FFAB session by community trust coaches. Data on retention to the programme were gathered in registers and reviewed by the researcher observing a given session.

Qualitative data collected included observations of session deliveries, interviews with participants and coaches, and focus groups with men participating in other initiatives at run by the community trusts. Members of the research team (CD, MP, KH, CG, HW, BB) observed 24 FFAB sessions across deliveries in three clubs. Observations were initially recorded in an unstructured form but were focused on the acceptability and fidelity of the programme content and delivery. For the final set of observations at Club E, observations were conducted using structured pro-forma allowing researchers (BB, HW, CG) to assess session delivery and participant engagement systematically (see Additional File 2). Semi-structured interviews were conducted with all FFAB participants after programme delivery ended (*N* = 13), with those who signed up but did not complete the programme (*N* = 1), with coaches involved in delivery (*N* = 9), and with other Trust staff involved with the programme (*N* = 3). All interviews were recorded and transcribed verbatim. Finally, qualitative focus group data were also collected in two focus groups with men who were currently taking part in other community trust programmes at Club D (*N* = 4) and Club E (*N* = 10). The focus groups explored how eligibility criteria, recruitment strategies and messaging might be adapted. These focus groups were audio recorded and transcribed.

As a thank you for participating in the research, by being observed, taking part in a semi-structured interview, or attending a focus group, participants were offered a ‘thank you’ high street gift voucher worth £20.

### Data analysis

We analysed data to assess the feasibility of the FFAB programme against the three progression criteria: recruitment, acceptability and fidelity. This was done using a mixed methods approach, specifically a convergent parallel design in which quantitative and qualitative data are analysed in parallel and then compared [33]. All quantitative data were analysed in Excel using descriptive statistics.

Observation and semi-structured interview data were analysed thematically in Microsoft Word following Braun and Clarke’s six stage approach, constructing themes inductively [34]. Initial coding was done by BB with support from HW, CB, GR. These researchers met regularly to compare coding decisions and discuss potential patterns identified in the data. Themes were constructed and refined collaboratively, using the coding, and then reviewed by the full research team to enhance rigour. The final thematic framework can be found in Table 4.

**Table 4:** Key themes and corresponding methods

Theme	Data Collection Methods
Recruitment	Participant interviews, Coach Interviews, Focus Groups, Observations
Programme Acceptability	Participant interviews, Coach Interviews, Focus Groups, Observations
Need for Programme	Participant interviews, Coach Interviews, Focus Groups
Gambling Sponsorship in Football	Participant interviews, Coach Interviews, Focus Groups, Observations
Stigma	Participant interviews, Coach Interviews, Focus Groups, Observations
Localisation	Participant interviews, Coach Interviews, Focus Groups, Observations

Focus group data were assessed using rapid analysis [35]. BB coded transcripts using a structured template, which was reviewed by additional members of the team (HW). The analysis informed the development and refinement of eligibility criteria, recruitment procedures and messaging, as the study moved from phase 1 to phase 2, and also contributed to the wider feasibility analysis.

The triangulation protocol technique [36] was used to structure our mixed methods assessment of programme feasibility. The research team systematically compared findings from the analysis of multiple quantitative and qualitative datasets, where relevant. Through this process, researchers reviewed findings from multiple datasets in relation to the three progression criteria, identifying instances of convergence and divergence to determine whether of the criteria were met. The use of multiple datasets, following the triangulation protocol technique, enhanced the credibility of the final assessment of FFAB’s feasibility.

### Ethics and informed consent

Ethical approval was obtained from the College of Social Sciences ethics committee at the University of Glasgow (Ref nos. 400190018 and 400210110). Participants and coaches provided informed consent after reviewing a detailed participant information sheet outlining the project’s objectives and their roles. The information sheet, including details on Behaviour Change Techniques (BCTs) such as SMART goals and if/then planning, also highlighted opportunities for engagement with partners like Beacon Counselling Trust and support services such as GamCare. Throughout enrolment, participants had opportunities to ask questions, ensuring ongoing informed consent. Coaches provided consent during dedicated training sessions and maintained it through regular communication with the research team. Consent procedures for interviews and focus groups were consistently rigorous, with researchers transparent about their roles during FFAB session observations.

## Results

Throughout the results section, we report qualitative data using pseudonyms.

### Feasibility of recruitment and retention

#### Recruiting clubs

The research design required that we recruit two clubs for each phase of the feasibility study. In both Phase 1 and Phase 2, however, we had to recruit three clubs in order to complete the study. Phase 1 clubs were labelled Clubs A, B and C; Phase 2 clubs were labelled Clubs D, E and F. During Phase 1, an additional club was required when Club A withdrew before moving to programme delivery and shortly before publicly announcing a change in sponsorship to a gambling organisation. In Phase 2, Club D was unable to recruit a group to take part in the programme and was replaced by Club F. As a result, we were able to recruit 6 clubs, but two withdrew.

#### Recruiting men

The study aimed to recruit a total of 40–60 eligible men, with 10–15 per club, spread evenly over the two phases. Throughout the recruitment process, the research team worked with participating clubs to track the recruitment materials and approaches used. Table 3 outlines the actions taken by each club to recruit men to the programme. With the exception of Club A, each participating community trust pursued recruitment across a range of media, utilising multiple channels and resources available to them. Direct endorsement by clubs (as opposed to Club Community Trusts) was rare. Notably, only Club F amplified the community trust-led recruitment efforts via its social media. Club E prohibited the research team from participating in match days: no leafleting at the ground, no match day programme advertising nor any other kind of advertising at the ground.

Despite the substantial range of recruitment attempts made by five of the six clubs, FFAB did not recruit sufficient participants to meet the recruitment progression criteria (See Table 5). Across the two phases, FFAB attracted 59 expressions of interest from men, of which only 30 were eligible to participate. All ineligible participants exceeded the PGSI cutoff. Participants in FFAV were all male, white and between the ages of 18 and 55.

**Table 5:** Recruitment and retention overview

	Expressed interest			Session attendance		
	Eligibility not known	Ineligible	Eligible for FFAB	Attended first session (% of eligible)	Attended four or more sessions	Attended final session
Phase 1						
Club A: Approach 1: PGSI < 8	0	5	0	N/A	N/A	N/A
Club B Approach 1: Approach 1: PGSI < 8	0	8*	9			
Club B Approach 2: PGSI score < 15	0	1	3			
* Club B: total*	0	*9*	*12*	*3* (25%)	*4*	*4*
Club C: Approach 2: PGSI score < 15	0	5	9	5 (56%)	1	1
Phase 2						
Club D: Approach 2: PGSI score < 15	2	3	0	N/A	N/A	N/A
Club E: Approach 2: PGSI score < 15	1	0	5	5 (100%)	5	5
Club F: Approach 2: PGSI score < 15	1	3	4	2 (50%)	N/A	N/A

##### Phase 1

During Phase 1, two different PGSI eligibility criteria were tested. Initially, the first approach targeted individuals with a PGSI score of less than 8, aligning with the programme’s goal of early intervention (referred to as ‘Approach 1’ in Table 5). However, data from Clubs A and B revealed that more than half of the interested men (*n* = 14) had a PGSI score higher than 8, highlighting a different risk profile than anticipated during the design phase.

After discussions with lived experience collaborators and project clinical advisors (including Beacon Counselling Trust), the eligibility criteria were expanded to include those with a PGSI score of 14 or less. This adjustment aimed to address a higher degree of gambling problems without targeting the most severe cases, balancing the ethical considerations of meeting a clear need and interest in FFAB, while prioritizing safeguarding measures. Upon implementing this change (Approach 2) in Clubs B and C, 21 men were screened as eligible to participate in the FFAB programme. Club B recruited 12 eligible men, meeting the target of 10–15 men, Club C recruited 9, falling short of the target by one participant.

##### Redesigning recruitment materials and processes

Following a review of qualitative data gathered in Phase 1, the study team identified recruitment as an area that needed improvement ahead of Phase 2, in order to recruit higher numbers of eligible men. Two focus groups were convened with men participating in other programmes at Clubs D and E to critically review recruitment materials and suggest alternative approaches.

In Phase 1, the programme was branded as “Reclaim the Game” with a tag line specifying that clubs were looking for men who wanted to reduce their gambling to take part in a structured support programme. Focus group participants critiqued the “Reclaim the Game” branding in three ways. First, they thought “Reclaim the Game” was easily confused for an existing campaign for fan ownership of clubs. Second, there was confusion over its applicability, as one participant stated, “*Well*,* what are you really reclaiming it from?”.* Third, the incongruence of clubs advertising a programme to reduce gambling whilst being sponsored by gambling companies was raised. Responding to these points, for Phase 2, the programme was rebranded as “Football Fans and Betting”.

Participants also suggested that the information included on recruitment materials and on social media was not specific or clear enough about what the programme involved. As one participant reviewing the project website said, “*From that I don’t know what it is or what taking part involves*,* so I wouldn’t signup*”. In response to these points, recruitment material was redesigned with a sharper focus on what the programme focussed on and what it involved.

##### Phase 2

In Phase 2, despite intensive recruitment efforts and changes in recruitment materials and strategies, the number of men expressing interest in the programme was low (see Table 5). At Club D, only five men expressed an interest in joining the programme. Three were screened out due to high PGSI scores. A further two men decided before screening that they no longer wanted to join the programme. As no eligible men were identified in Club D, the FFAB programme did not run at all in this club.

In Club E, after an unsuccessful recruitment drive, the coaches identified 5 men in an existing programme that were interested and eligible to take part in the programme. The programme was run at a local hub by the Community Trust as a ‘bolt-on’ to an existing social football group, and retention was good. All five men attended the first, fourth and final sessions.

At Club F, eight men signed up. One sign up was unreachable after signup. Three were ineligible due to having PGSI scores > 14. Four were eligible. However, in Club F, at the point of the start of the programme, only two out of four men indicated that they were still able to attend the programme. As a result, the decision was made not to proceed.

#### Making sense of recruitment challenges

While FFAB came close to meeting recruitment targets in Phase 1, in Phase 2 recruitment was considerably worse. Our qualitative dataset shed some light on factors that contributed to this outcome. Firstly, across interviews and observations, men and coaches suggested that FFAB was unable to connect with its target audience because the target audience did not view the programme as ‘for them’. For example, during observations at Club E, captured in field notes, participants referenced more than once how one man had left a sign-up session with the coaches after five minutes saying that FFAB was ‘not for him’. The men commented that they felt he “needed” it more than any of them based on their knowledge of his frequent betting and losses. This example illustrates the perception that attending a programme like FFAB was ‘for’ someone who was experiencing more obvious or severe harms.

Coaches also commented on this and brought in the additional issue of stigma. As one coach, Sam, suggested:

I think there’s the stigma. I think again, the people that this was designed for being in that sort of lower end of the scale [PGSI] in terms of it being problematic to their life, I don’t know whether there’s something in that around denial. Do people see it as an issue at that point? Does it only become an issue to people when it’s a real issue? [Sam, Trust manager interview, Club F]

Here, Sam is suggesting that low recruitment to FFAB could be explained by men in the target group viewing programmes as ‘for’ those who ‘have a problem’ and that ‘having a problem’ is stigmatised.

An additional factor raised by participants across the qualitative dataset was the perceived incongruence between a football club backing FFAB but, at the same time, receiving substantial funding from gambling companies:

On the topic of sponsorship of football by gambling companies, one participant questioned how committed Club E was to FFAB – “they are putting on this programme but also promoting their betting sponsors alongside” – so they queried the disconnect. The coaches explained that the Trust are different from the Club and there’s not much they can do about the Club’s actions. A different participant also mentioned one of the first team chatting about betting a lot too. [Session 1 observation, Club E]

This tension likely played a role in the difficulties we had recruiting to the FFAB programme.

While these factors and the data that underpins them are only part of the explanation, it is likely that perceptions of who FFAB is ‘for’ and the stigma attached to attending a programme interpreted as addressing a ‘problem’ had linked, negative impacts on recruitment that were compounded by inconsistency in the perceived relationship between clubs and gambling companies.

Our analysis of recruitment related data understandably highlights the challenges FFAB faced, it also pointed to a factor which seemed to support recruitment. Club B recruited the highest number of eligible men. One of the coaches explained that they had targeted their efforts within a 10 km radius of the club, focussing on local community members.

### Fidelity

Low recruitment numbers contributed to challenges delivering FFAB as intended. Table 5 shows the number of participants attending the first and last sessions at each club and the number who attended at least half of the 8 sessions. Across the four deliveries of FFAB included in this feasibility, none delivered the programme to a group of the intended size i.e. 10–15 men. A group of 10–15 participants was intended to foster and model social support for behavioural change across the entire programme. Beyond this, and as documented in Additional File 1, eight specific activities in the programme were intended to explicitly draw on social support from the group, and beyond, to achieve the intended behaviour change. These observations indicate that FFAB could not be delivered ‘with reasonable fidelity’.

### The acceptability of FFAB to coaches and participants

Despite challenges in recruitment and retention, both of which compromised fidelity, ten out of sixteen men who enrolled in the FFAB program during its initial phases successfully completed the program. Feedback from these participants and the coaches who delivered the sessions was notably positive. In this section, we present an evaluation of acceptability by exploring: the perceived need for intervention, views on the content and format of the programme, the social enjoyment of attending the programme, the changes it led to and how others were impacted by the programme. Through this analysis, we demonstrate that FFAB was acceptable to those who delivered and participated in FFAB.

#### Intervention need

Across the observation and interview data, there was consensus among both participants and coaches that intervention to prevent and reduce gambling harms among men is needed. For example, a participant at Club E who had faced severe gambling harms in his recent past, suggested that: “I think I wouldn’t have ended up doing what I did then if I’d had a programme like this.” [Robbie, participant interview, Club E] Robbie’s view, then, is that the programme would have prevented him from experiencing gambling harms. However, he noted in the same interview that the programme had still been useful in his recovery.

Coaches also reported that FFAB was addressing a perceived need, for example:

It’s like the same with a lot of programmes and projects that we that we run here. They are needed. I feel like it’s a needed project. It’s the same with like men’s mental health. It’s a case of these sessions are needed to help people for the better. [Josh, coach, Club F]

So, while FFAB was unable to attract feasible numbers of participants, those who attended and delivered the programme saw considerable value in the attempt to intervene in gambling harms among men.

### Programme content and delivery

Throughout the interviews, participants and coaches described finding the content and delivery of the programme acceptable. For example, one participant said:

Eh yeah, it was actually, like, interesting. And not boring like school. It wasn’t as much like school as I thought. (Calvin, participant interview, Club E)

This corresponded with comments from other participants, who told us that the programme had been engaging and informative in a way that kept them coming back each week.

Participants commented that the coaches were critical to their experience of FFAB:

I think every week I got a sense of achievement, even if it…even in me bad week, do you know, like, even when I was at worst in that week, [Coach] still found ways to make me feel like I’ve still achieved. [Paul, participant interview, Club C]

In some instance, coaches extended their support for participants by making informal connections, outside of the programme. For example, during interview, one participant praised a coach, as they had supported his son to access a football programme. From observations, the research team also recorded notes reflecting a feeling that the coaches were key to delivery of the programme in how they built relationships with participants, generated rapport and facilitated the sessions.

A key element of the FFAB programme was specifically designed to help participants better understand the ways in which the commercial gambling industry operates and how this industry makes its profits. This focus was embedded throughout the programme and covered in detail in a specific session focusing on industry tactics. There was support among participants for this approach.

An example of this support relates to how content on advertising and marketing strategies used by the gambling industry captured the sustained attention of participants. For example, one participant said:

Every time I watch the football now, I look at it and I think there’s one [a gambling advert] there, there’s one there. You know what I mean? [Jamie, participant interview, Club E]

There were similar examples of participants noticing the presence of gambling operators and advertising in their everyday lives. For example:

One participant said his homework [*which he had set himself*] this week had been to identify who it is that he has seen advertising on phone boxes locally. Turns out it is “Roxy bingo”. A few of the guys recognised this and discussed the specifics of the adverts which apparently also feature a “bowling ball”. Showing brand recognition. [Session 4 observation, Club E]

These examples illustrate how men taking part in FFAB took their learning from the programme into their daily lives and suggests an increased awareness of the visibility and tactics of gambling operators.

#### Social Enjoyment of FFAB

While the group sizes across the four deliveries did not reach the target of 10–15 men, participants still emphasised the importance of social connection through FFAB. Specifically, participants reported enjoyment of the social interaction during FFAB sessions. For example, being able to connect with other men who had undergone similar experiences:

I mean the other lads in the same boat, to say…like they were all wanting to do the same as me. It wasn’t people just wanting to cut down, people all wanted to stop, so it was like [pause], it’s like, yeah, just testing yourself basically and seeing if you can do it. (Stan, participant interview, Club B)

Thus, meeting likeminded men and having a supportive group where their experiences of stopping gambling could encourage each other was seen as useful. Notes from observations detailed an array of comments describing how the men had enjoyed growing closer to each other during the programme. For example, a fieldnote from the final session at one club noted,

A raucous conversation then breaks out with different people detailing the funniest moments they remember from preceding weeks. Many revolve around the missing participant today… Someone finally breaks this tangent a bit, “Thing is with these discussions, like, we’ve learned about each other, haven’t we?” Alluding to the social element of the programme. There’s agreement in the group. [Session 8 observation, Club E]

#### Changing approaches to gambling

All participants who attend FFAB reported benefitting from the programme when interviewed. This was particularly true of those who had identified a desire to significantly reduce their betting. For example, one participant at Club B described his experience of completing FFAB:

Like I was changed in myself, the way I think about things and the way, um, I’ve actually gone out, gone to the gym, I’m more open with my wife about like the finances and the ways I’m planning ahead with the holidays, making sure there’s a difference, like I’ve definitely changed the way I do things. [Stan, participant interview, Club B]

Through accounts such as Stan’s, participants described how the FFAB programme enabled them to reposition gambling and its impacts in their lives. They de-prioritised gambling, pursued other activities, were less secretive about financial matters and improved their relationships.

The exit interviews with participants from Club E suggested that some attributed a reduction in their gambling to the programme, irrespective of the strength of their intention to reduce their gambling when joining. For example, in the final session at Club E, one of the men who reported regularly putting on small accumulator bets on the weekend’s football fixtures had not put a bet on for the duration of the programme as FFAB had made him reconsider this routine.

#### The social life of FFAB

Some of the coaches and participants described passing on what they had learned from FFAB into their personal networks. For example, a coach from Club 5 described giving his brother-in-law the challenge of counting gambling adverts whilst watching the boxing:

So, when I told him what it was [the impact of gambling advertising], he asked me so why is it advertised a hundred times around the ring?! I went to him; count how many times you see betting until the next advert. He said he saw it like 10 times just whilst the boxers were in the ring. [John, Coach interview, Club E]

One of the coaches also noted that he had decided to stop betting himself because of what he had learnt from the programme.

Several participants mentioned that they had spoken to others in their social networks about the programme. One highlighted how seeing the advert for FFAB had prompted him to talk to his wife about his gambling for the first time, and how his wife encouraged him to attend. However, several participants described being uncomfortable talking about the programme to people outside of the programme setting. For example, one participant commented that despite not gambling much himself (he joined the programme to support a friend who he felt would benefit and to get to know the group better) he had been guarded about the details of the programme and his involvement to others:

Researcher: Have you talked to anyone not on the programme about it or anything that was in it?

Rob: No, not really. We all speak amongst it ourselves [meaning the group]. Whoever was in there, that’s really who we speak to, because if I tell someone I’m going to a gambling project, and they’re like ‘Ohhh!’ [does this with a tone of suspicion and disgust and then laughs]. (Rob, participant interview, Club E)

We see from this the way in which stigma functioned even for those who joined the programme. The potential of learning from or enthusiasm for the content of the programme being spread outside of the group taking part was limited because participants felt they would be stigmatised by virtue of being involved in the first place.

## Discussion

FFAB was launched in six clubs. Despite multiple attempts to refine and improve recruitment processes, no club was able to recruit the target number of participants to the programme. Recruitment challenges had a knock-on impact on fidelity, with no club in the feasibility delivering the programme to the intended group size. It is clear, then, that the FFAB programme was not feasible on the grounds of recruitment and fidelity, though it was acceptable to those who participated. The fact that the study took place following the end of COVID-19 distancing restrictions is likely to have impacted the study in a range of ways.

COVID-19 aside, the programme faced two key challenges that appear to be driven by broader contextual factors: the pervasive commercial entanglement between football and the gambling industry, and persistent stigma surrounding gambling harms. In relation to the former, our analysis clearly shows considerable scepticism among participants targeted at the extent to which their clubs were well placed to offer FFAB when taking sponsorship monies from the industry. This scepticism is arguably supported, at least in part, by the evidence we presented relating to: Club A’s sudden withdrawal due to a forthcoming new sponsorship contract with a gambling company; Club E preventing the research team from accessing their stadia on matchdays to support recruitment efforts; and all clubs (except Club F) refusing to amplify recruitment efforts through club social media. These instances highlight the challenges of working in an environment in which the commercial influence of the gambling industry runs throughout the landscape of football, and impacted, in various ways, on our recruitment efforts.

Stigma emerged as a recurring theme across focus groups, interviews, and observations, echoing concerns raised in the wider literature about the challenges of help-seeking among people experiencing gambling harm [37, 38]. Participants commonly perceived FFAB as “for men with a problem,” a framing that likely prevented potential participants from engaging with FFAB. In this way, then, the FFIT model as applied here, was unable to recreate the de-stigmatising effects it achieved for male weight loss in the context of gambling harm reduction for men. In this way, then, the model elicited a different, unsuccessful, interaction between intervention and context [39].

Few other studies have taken a group-based approach to preventing gambling harms among men. Indeed, prevention research in this demographic tends to focus on individual-level intervention in the practice of gambling e.g. pre-commitment/limit-setting, self-exclusion, removing ATMs from gambling venues [25], and places low levels of emphasis on socially-grounded mechanisms for change [26]. FFAB, by contrast, employed a range of intervention approaches with at least some evidence of efficacy [2], delivered in a social setting intended to potentiate efficacy. While too few participants were recruited to begin to draw inferences about potential efficacy, it is clear that participants found FFAB groups to be socially supportive and enjoyable environments, as reported in the FFIT study [30]. In the absence of a full and faithful delivery, and provided recruitment challenges can be overcome, the novel group approach taken in FFAB remains worth testing.

Our evaluation suggested that one potential route to improved recruitment, and thus to enable better assessment of the content of the intervention and its potential for efficacy, is to adopt a more localised approach. Where local-level relationships with coaches and delivery hubs were well established, clubs were more successful in engaging participants. These findings resonate with literature on early intervention and community-based health promotion, where trust, familiarity, and accessibility are critical enablers of uptake [40].

However, a shift toward more localised approaches also raises questions of scalability, equity, and resource allocation. If future versions of FFAB or similar interventions are to adopt this model, careful planning will be needed to support delivery infrastructure, training, and sustainability.

### Strengths and contributions

To our knowledge, this study represents the first feasibility trial of a gambling harm prevention programme for at-risk male sports bettors delivered in a professional football setting. It contributes new insights into the unique challenges of engaging this population and adapting an otherwise successful model of recruitment and retention from other programmes. By documenting both the barriers and enablers encountered, the study advances our understanding of how gambling harm prevention might be better tailored to male sports fans.

### Limitations

A key limitation of the study is that it did not reach the target number of participants. As a result, FFAB was neither delivered nor evaluated in the intended group sizes, which may have affected dynamics and outcomes. The study would also have benefited from collecting data on: participants’ expectations versus their actual experiences of the programme and the possible impact of programme rebranding on recruitment and participants’ perceptions. Nevertheless, the use of a multimethod design - combining qualitative interviews, focus groups, and observational data - offers a strong foundation for interpreting these findings and guiding next steps.

### Implications for policy and structural change

The findings suggest that football clubs’ commercial relationships with the gambling industry directly constrained their willingness to support recruitment for the intervention through official channels. With the exception of Club F, this lack of institutional support reinforced norms of silence and avoidance surrounding gambling harm, signalling how commercial interests can override opportunities to confront a stigmatised issue. In this context, the clubs’ inaction represented a missed opportunity to challenge stigma and demonstrate social responsibility. Although the study was not designed to analyse gambling marketing per se, the data reveal how the pervasive influence of gambling sponsorship within football can undermine public health initiatives that seek to prevent or reduce harm. For policymakers, these findings highlight the structural barriers created by industry partnerships and the importance of developing strategies that enable sporting organisations to engage with gambling harm without being constrained by commercial ties.

### Future research directions

While FFAB did not function as a scalable health intervention, its acceptability among participants and coaches points to the potential for adaptation. Future research should explore FFAB’s use as a recovery support or signposting mechanism for high-risk individuals, provided appropriate safeguards are in place. Efforts should also focus on understanding how best to integrate destigmatisation work and structural change, particularly in settings where commercial influences run counter to public health aims. Recruitment should be grounded in local networks that are independent of the gambling industry and trusted by community members. However, additional research is required to understand when and how particular social interactions and local contextual features support, or conversely, impede the effective implementation of prevention programmes. As gambling-related harm is not abating, future interventions must take seriously both the socio-cultural dynamics of gambling and the systemic forces that drive it.

## Conclusion

This study found that FFAB is not feasible in relation to recruitment and fidelity criteria but was acceptable to those who participated in and delivered the programme. The relationship between football clubs and the gambling industry, as well as the stigmatised nature of gambling harms, were identified as key barriers to recruitment. Where the programme was delivered, participants achieved meaningful changes in their lives that likely reduced gambling harms. As we pursue effective strategies to prevent gambling harms among men, more work is required to develop selective and targeted public health interventions that support the much-needed universal interventions that policy action can deliver.

## Supplementary Information

Supplementary Material 1.

Supplementary Material 2.

Supplementary Material 3.

Supplementary Material 4.

Supplementary Material 5.

## Data Availability

This is a qualitative study and therefore the data generated is not suitable for sharing beyond that contained within the manuscript. Further information can be obtained from the corresponding author.

## Abbreviations

BCTs: Behaviour Change Techniques
FFAB: Football Fans and Betting
FFIT: Football Fans in Training
PGSI: Problem Gambling Severity Index
