# Supplementary material for: An uneven playing field: a mixed methods, multiphase feasibility study of a programme to reduce gambling among at-risk men in a professional football club setting
Source: BMC Public Health. 2026 Mar 5;26:1565. doi: 10.1186/s12889-026-26845-z (PMC13188234; doi:10.1186/s12889-026-26845-z)
Supplement: Supplementary file 1 — Supplementary Material 1. [file 12889_2026_26845_MOESM1_ESM.docx]

**Football Fans and Betting (FFAB) Feasibility Study**

**Indicative Interview Topic Guide – Semi-structured post-programme Coach Interview**

**Introductions and Thanks**

- Brief welcome, overview of the purpose of the interview and expected duration (45-60 minutes) – We are looking to ask you about your experiences of delivering the Reclaim the Game programme to men aged 18-44 so we can find out what you thought about the programme and the resources (coach notes, Reclaim the Game app). We will use your suggestions to further improve the Reclaim the Game programme in future.
- Anything you say is important to me and the other researchers so please don’t be afraid of speaking your mind.
- I will audio-record the discussion, and the recordings will be kept for 10 years after the project finishes, but everything you say will be treated in the strictest confidence; all names mentioned will be changed for publication/presentation purposes.

**Discussion points**

First, can you tell me a bit about the Reclaim the Game programme you delivered?

Which club? Date and time? Why were these times chosen?

How many men were involved?

What was attendance like over the 8-weeks?

What did you think were the main things that the Reclaim the Programme was trying to achieve?

**Reflections on the initial coach training**

I would like to ask you a bit about the initial training you received.

How did you find the initial coach training session(s)? Prompt: Were they helpful/not helpful? Was there anything that could have been done differently?

How did you find the content on gambling harms and safeguarding?

How did you find the mock session deliveries?

How did you feel about the behaviour change elements related to gambling specifically (e.g. goal setting, self-monitoring and planning)?

**Reflections on recruitment**

I would like you talk about the recruitment strategies used to recruit men onto the programme. Please talk me through the strategies that you employed.

How were these approaches similar to other programmes you have recruited previously? Prompt: What was similar/different?

What did you feel were the most effective strategies?

What did not work so well?

How did you adapt the materials/approaches?

What do you think you would do differently in future?

What else do you think could be done to facilitate recruitment to this programme in future?

What did you think about the sign up process?

What did you think about Reclaim the Game website?

**Reflections on the Reclaim the Game programme delivery**

Now let’s talk about your experience of delivering the Reclaim the Game programme. Overall, how did you feel it went?

Did you find anything particularly helpful in enabling you to deliver the programme? Prompt: Coach manual, PowerPoint slides – did you find these useful? Why?

How useful did you find the WhatsApp group to communicate with the men between sessions?

How did you feel about the group involvement during sessions?

Was there anything that made the delivery of the programme challenging? Prompt: Group dynamics/size, age range, disruptive personalities, different levels of fitness or abilities, access to facilities?

How did you feel about dealing with the questions the men asked? Prompt: Were there any difficult topics or issues you felt that you couldn’t address or discuss? Was there anything of particular concern? If so, how confident did you feel addressing these issues?

How did you find the support you were given to deliver the programme?

**Reflections on the Reclaim the Game programme content**

What did you think was good about the programme?

What did you think was not so good about the programme? Do you have any suggestions for improvement?

Which parts of the programme did you think were effective in helping the men to reduce their gambling activity? Why?

Were there any components of the programme that you felt were not useful? Why? Would you remove these elements or change them? Prompt: The Reclaim the Game app, SMART goal setting or other activities in the classroom elements, the group exercise sessions

How important do you think the physical activity/exercise components of the programme are?

A key focus of Reclaim the Game was to help the men to make long term changes to their gambling, not just make changes that could not be maintained beyond the 8 weeks. Which parts of the programme do you think were particularly helpful in trying to achieve this? Do you think the programme was effective in achieving this? Why/Why not?

**Future changes to the programme going forward**

Are there any changes that you would like to see made to any particular elements of the programme? Prompt: More information, more training/materials, targeting of different men? Why?

How would feel about opening the programme up to include a broader range of men, for example, extending the age range from 18-44 to 18-65 years? How would you feel about including men who may have experienced more severe gambling issues?

At end of discussion, summarise what has been said and ask if there is anything else they would like to add or if there is anything else we should consider for improving the programme in future.

Thank them again for all of their input in developing the Reclaim the Game programme.
