# Supplementary material for: An uneven playing field: a mixed methods, multiphase feasibility study of a programme to reduce gambling among at-risk men in a professional football club setting
Source: BMC Public Health. 2026 Mar 5;26:1565. doi: 10.1186/s12889-026-26845-z (PMC13188234; doi:10.1186/s12889-026-26845-z)
Supplement: Supplementary file 3 — Supplementary Material 3. [file 12889_2026_26845_MOESM3_ESM.docx]

**Football Fans and Betting (FFAB) Feasibility Study**

**Indicative interview topic Guide – Semi-structured Exit Interview**

**Introductions and Thanks**

- Brief welcome, overview of the purpose of the interview and expected duration (45-60 minutes) – We are looking to ask you about your experiences of the Reclaim the Game programme and find out what you thought about the programme, and why you stopped attending the weekly sessions. We will use your suggestions to further improve the Reclaim the Game programme in future.
- Anything you say is important to me and the other researchers so please don’t be afraid of speaking your mind.
- I will audio-record the discussion, and the recordings will be kept for 10 years after the project finishes, but everything you say will be treated in the strictest confidence; all names mentioned will be changed for publication/presentation purposes.

**Discussion points**

First, can you tell me a bit about your participation in the Reclaim the Game programme: Which club did you attend? What time did the programme run? Who were your coaches?

**Your experience of Reclaim the Game**

Now let’s talk about your experience of taking part in the Reclaim the Game programme.

How many of the weekly sessions did you attend? Prompt: Why did you stop coming along to the sessions?

Was there anything that could have been done to encourage you to continue with the programme?

Were there any aspects of the programme that you thought were unnecessary or didn’t’ like? If so, which parts and how could we change them?

Was there anything else you would have liked to have been included in the 8-week programme? What about the length of the sessions/course?

Which sessions did you find most useful/interesting? Why?

Do you feel that the Reclaim the Game had any positive impacts on your life? If so, what was it about the programme that helped you experience these? Prompt: role of the group; coaches; information/key sessions; tools (including SMART goal setting and self-monitoring of time and money spent gambling; physical activity; Reclaim the Game app)

What did you think about community coaches connected with [name of club] delivering the programme? What did you feel about their general approach with the group? [If they say ‘great’ or equivalent – can you tell me a little more about why they were great].

What did you think about the physical activity part of the sessions? What did you like about them? What did you not like, if anything? Did you usually exercise or regularly do some form of physical activity prior to taking part in Reclaim the Game? (What? How much?)

Did you talk to anybody else about the programme, if so, who and what did you say about it? If not, why did you not talk about the programme?

What sorts of reactions have you had from family and friends about you taking part in Reclaim the Game? (Do they know?)

Who do you think could benefit most from a programme like Reclaim the Game? What would be the best ways to reach out to involve these people? Do you think we are currently trying to reach the right people?

How would you feel about the programme being widened to include a broader range of men, for example, extending the age range from 18-44 to 18-65 years?

How would you feel about involving men who may have experienced more serious gambling issues? Prompt: If the programme were to be extended to men with serious gambling problems, what do you think we should do to keep people safe?

**Prior to joining Reclaim the Game (if time ask)**

How did you hear about Reclaim the Game? Prompt: social media (including Facebook, Instagram, Twitter), club/community trust website, match day advert, poster/flyer, local press – probe in case of multiple sources.

Do you think we could have done anything better to attract participants?

What if anything do you think may put people off responding to this type of advertising and marketing?

What did you think about the Reclaim the Game website and signup process? Are there any parts of this process that you think might have put other men off? Which parts?

Can you tell me why you wanted to take part in the Reclaim the Game programme at [name of club]? What did you hope to achieve? How important was the affiliation with the football club?

Thinking back to before you started Reclaim the Game, how did you feel about coming along to the very first session? Probe to explore thoughts, expectations, feelings, nerves, excitement, etc., and reasons for these.

Had you ever attended other services or support for gambling prior to signing up to take part in Reclaim the Game?

At end of discussion, summarise what has been said and ask if there is anything else they would like to add or if there is anything else we should consider for improving the programme in future.

Thank them again for all of their input, and remind of sources of information and additional support (Beacon Counselling Trust).
