# Supplementary material for: An uneven playing field: a mixed methods, multiphase feasibility study of a programme to reduce gambling among at-risk men in a professional football club setting
Source: BMC Public Health. 2026 Mar 5;26:1565. doi: 10.1186/s12889-026-26845-z (PMC13188234; doi:10.1186/s12889-026-26845-z)
Supplement: Supplementary file 4 — Supplementary Material 4. [file 12889_2026_26845_MOESM4_ESM.docx]

# Additional File 1: Overview of Programme Sessions mapped to Behaviour Change Techniques per activity

| **Session** | **GIST Category** | **BCT Taxonomy** |
| --- | --- | --- |
| **Session 1** |  |  |
| *Session 1 Aims* |  |  |
| 1) Encourage the men to get to know each other and feel at ‘ease’ 2) Facilitate social bonding within the group, through their shared love of the game, a shared opportunity to do physical activity sessions (have a ‘kick about’) led by club coaches, and to learn from club coaches’ expertise about the benefits of physical activity for general health, wellbeing, and mental health  3) Provide an overview of the programme, including an introduction to using the app and the setting up shared social media to communicate with each other  4) Encourage the men to reflect on their current relationship between football and betting and how this influences their daily lives |  |  |
| ***Activities*** |  |  |
| 1.1. Welcome and introduction (5 mins) | Motivational Enhancement | Verbal persuasion about capability |
| 1.2. Football focus icebreaker (10 mins) | Plan Social Support | Social support (unspecified) |
| 1.3. Overview of programme (10 mins) | Motivational Enhancement | Verbal persuasion about capability, Commitment |
| 1.4. Gambling industry icebreaker (10 mins) | Information Gathering | Information about antecedents, re-attribution |
| 1.5. Introduction to App (10 mins) | Self-Monitoring | Self-monitoring of behaviour, Self-monitoring of outcome(s) of behaviour, Adding objects to the environment |
| 1.6. Setting up social media group (10 mins) | Plan Social Support | Social support (practical), Adding objects to the environment |
| 1.7. Tour of stadium | Planning Social Support? | Behavioural practice/rehearsal, habit formation, social support (unspecified) |
| 1.8. End of session (5 mins) |  |  |
| **Session 2** |  |  |
| *Session 2 Aims* |  |  |
| 1) Understand facts around what is and is not gambling, including awareness of risk and bias  2) Reflect on the role that the gambling industry advertising and marketing can have on influencing gambling behaviour as well as the importance of being aware of their own actions  3) Be clear on how to use SMART goals to assist them in making small, achievable steps towards reducing their gambling behaviour |  |  |
| ***Session 2 Activities*** |  |  |
| 2.1. Welcome back, Review of Week 1 | Problem solving (for PA) | Verbal persuasion about capability. Problem solving. |
| 2.2. What is gambling activity? | Information provision | Information about antecedents, re-attribution |
| 2.3. Gambling advertising and marketing | Information gathering, information provision | Information about antecedents, re-attribution |
| 2.4. Locus of control exercise | Cognitive restructuring | Re-attribution |
| 2.5. Introduction to SMART goal planner | Self Monitoring, Information provision | Self-monitoring of behaviour (Gambling), Instruction on how to perform a behaviour, Adding objects to the environment |
| 2.5. Reviewing steps and pitch side activity | Behavioural substitution | Behaviour substitution, Behavioural practice/rehearsal, Habit formation, Instruction on how to perform a behaviour, Review behaviour goals, Goal setting, Self-monitoring of behaviour, Feedback on behaviour |
| **Session 3** |  |  |
| *Session 3 Aims* |  |  |
| To explore motivations for, and context of betting, and for the men to review and refine their goals to help reduce their gambling behaviour.  Main aims are to: 1) Review SMART goal setting, discuss any problems encountered, and explore ways of overcoming possible obstacles or challenges  2) Recognise the reasons underpinning why people gamble so the men can reflect on their own motives and identify potential triggers |  |  |
| ***Session 3 Activities*** |  |  |
| 3.1. Welcome back, Review of Week 2 |  |  |
| 3.2. Reviewing SMART goals | Self Monitoring, Feedback, Problem solving, Information gatherting | Self-monitoring of behaviour (PA), Feedback on behaviour, Problem solving, Information about antecedents, re-attribution, Reduce negative emotions |
| 3.3. Why people gamble | Information gathering, Information provision, Decisional balance | Information about antecedents, re-attribution, Information about health consequences, Pros and cons |
| 3.4. Getting additional help |  |  |
| 3.5. Review of PA/fitness/general wellbeing and introducing strength and balance guidance and pitch side activity | Behavioural substitution | Behaviour substitution, Behavioural practice/rehearsal, Habit formation, Instruction on how to perform a behaviour, Review behaviour goals, Goal setting, Self-monitoring of behaviour, Feedback on behaviour |
| **Session 4** |  |  |
| *Session 4 Aims* |  |  |
| 1) Enable the men to better understand how gambling and betting companies make their money 2) Facilitate reflection on what gambling and betting companies do with the data they have on consumers 3) Provide an overview of the different tools available to support men to stick to their goals |  |  |
| ***Session 4 Activities*** |  |  |
| 4.1. Welcome back and review of Week 3 SMART targets (15 mins) | Goal setting, Self-monitoring, Feedback | Goal setting, Self-monitoring of behaviour, Feedback on behaviour, Review behaviour goals, Habit formation |
| 4.3. Gambling Industry Quiz | Information gathering, Information provision, Decisional balance | Information about antecedents, re-attribution, Information about health consequences, Pros and cons |
| 4.4. What does the gambling industry do with your data? | Information gathering, Information provision, Decisional balance | Information about antecedents, re-attribution, Information about health consequences, Pros and cons |
| 4.5. Tools to protect yourself from gambling industry tactics | Goal setting, Relapse prevention, Reduce negative emotions, Plan social support | Goal setting, Action planning, Mental rehearsal of successful performance, Social support (unspecified) |
| 4.6 End of session recap | Goal setting |  |
| 4.7 Reviewing PA/fitness/general wellbeing and pitch side activity | Behavioural substitution | Behaviour substitution, Behavioural practice/rehearsal, Habit formation, Instruction on how to perform a behaviour, Review behaviour goals, Goal setting, Self-monitoring of behaviour, Feedback on behaviour |
| **Session 5** |  |  |
| *Session 5 Aims* |  |  |
| 1) To map men’s social influences on gambling, both positive and negative  2) To explore strategies that enable their social network to support change  3) To develop personalised IF/THEN plans to help identify risky situations and how to avoid them |  |  |
| ***Session 5 Activities*** |  |  |
| 5.1. Welcome back and review of Week 4 SMART targets (15 mins) | Goal setting, Self-monitoring, Feedback | Goal setting, Self-monitoring of behaviour, Feedback on behaviour, Review behaviour goals, Habit formation |
| 5.2. Dicussion of How do our social networks influence us? (25 minutes) | Information gathering, Problem solving, Plan social support | Information about antecedents, Problem solving, Social support (practical), Social comparison, Identification of self as role model |
| 5.3. IF/THEN plans (10 mins) | Goal setting, Relapse prevention, Plan social support | Goal setting, Action planning, Mental rehearsal of successful performance, Social support (unspecified) |
| 5.4. Review of steps and pitch-based exercise (30 mins) | Behavioural substitution, Self-Monitoring, Goal Setting | Behaviour substitution, Behavioural practice/rehearsal, Habit formation, Instruction on how to perform a behaviour, Review behaviour goals, Goal setting, Self-monitoring of behaviour, Feedback on behaviour |
| **Session 6** |  |  |
| *Session 6 Aims* |  |  |
| *1) Understand nature of gambling harms, in terms of the potential range of impacts and greater numbers of people affected. 2) Understand the different domains of harm, including for e.g., work, relationships, money  3) Encourage the men to reflect on the potential range of harms gambling may cause in their own lives 4) Encourage them to think through ways they might want to change their behaviour to reduce those potential / actual harms* |  |  |
| ***Session 6 Activities*** |  |  |
| 6.1. Welcome back and review of Week 5 SMART targets (15 mins) | Goal setting, Self-monitoring, Feedback | Goal setting, Self-monitoring of behaviour, Feedback on behaviour, Review behaviour goals, Habit formation |
| 6.2. Understanding gambling harms (20 mins) | Decisional balance, Information provision | Pros and cons, Salience of consequences, Information about social and environmental consequences, Information about emotional consequences |
| 6.3. Reflecting on harms (15 mins) | Motivational enhancement | Identity associated with changed behaviour, Information about emotional consequences |
| 6.4. Getting help (10 mins) | Plan social support | Social support (unspecified) |
| 6.5. Review of steps and pitch-based exercise (30 mins) | Behavioural substitution, Self-Monitoring, Goal Setting | Behaviour substitution, Behavioural practice/rehearsal, Habit formation, Instruction on how to perform a behaviour, Review behaviour goals, Goal setting, Self-monitoring of behaviour, Feedback on behaviour |
| **Session 7** |  |  |
| *Session 7 Aims* |  |  |
| 1) Review the men’s progress towards their goals 2) Understand that setbacks are normal when changing behaviour and remind them of the tools to help support them overcome potential setbacks/relapses/triggers in future  3) Establish how the men are feeling now that they are nearing the end of the programme and look out for anyone that may need further support |  |  |
| ***Session 7 Activities*** |  |  |
| 7.1. Welcome back and review of Week 7 SMART targets (15 mins) | Goal setting, Self-monitoring, Feedback | Goal setting, Self-monitoring of behaviour, Feedback on behaviour, Review behaviour goals, Habit formation |
| 8.2. Targets and goals self-reflection (15 mins) | Goal setting, Self-monitoring, Feedback, Relapse prevention, Problem solving | Verbal persuasion about capability, Goal setting, Review behaviour goals, Action planning, Self-monitoring, feedback, Action planning, Reduce negative emotions, Problem solving |
| 8.3. Review of steps and pitch-based exercise (40 mins) | Behavioural substitution, Self-Monitoring, Goal Setting | Behaviour substitution, Behavioural practice/rehearsal, Habit formation, Instruction on how to perform a behaviour, Review behaviour goals, Goal setting, Self-monitoring of behaviour, Feedback on behaviour |
| **Session 8** |  |  |
| *Session 8 Aims* |  |  |
| *1) Review the men’s progress towards their goals and identify important tools for maintaining change 2) Reflect on some of the key benefits experienced due to any changes made throughout the programme 3) Celebrate the men’s achievements and promote positive ethos to encourage sustained changes beyond the FFAB programme.* |  |  |
| ***Session 8 Activities*** |  |  |
| 8.1. Welcome back and review of Week 8 SMART targets (20 mins) | Goal setting, Self-monitoring, Feedback | Goal setting, Self-monitoring of behaviour, Feedback on behaviour, Review behaviour goals, Habit formation |
| 8.2. Locus of Control exercise & review of progress (15 mins) | Decisional balance, Goal setting | Pros and cons, Review of behaviour goals |
| 8.3. Benefits of change (5 mins) | Motivational enhancement | Verbal persuasion about capability, Identity associated with changed behavior |
| 8 4. Celebration of achievements / Graduation Ceremony (20mins) | Motivational enhancement | Verbal persuasion about capability, Identity associated with changed behavior, Social support (practical) |
| 8.5 The Match (35 mins) | Behaviour substitution |  |
