# Supplementary material for: An uneven playing field: a mixed methods, multiphase feasibility study of a programme to reduce gambling among at-risk men in a professional football club setting
Source: BMC Public Health. 2026 Mar 5;26:1565. doi: 10.1186/s12889-026-26845-z (PMC13188234; doi:10.1186/s12889-026-26845-z)
Supplement: Supplementary file 5 — Supplementary Material 5. [file 12889_2026_26845_MOESM5_ESM.docx]

**FFAB Session Observation Proforma**

**SESSION No. 6 Leicester_ : 25/07/23**

**Name of researcher:**

|  | | |
| --- | --- | --- |
| On a 1-10 scale, how satisfied were you with the content of the session overall? | Very unsatisfied  Very satisfied   \| 1 \| 2 \| 3 \| 4 \| 5 \| 6 \| 7 \| 8 \| 9 \| 10 \| \| --- \| --- \| --- \| --- \| --- \| --- \| --- \| --- \| --- \| --- \| \| ☐ \| ☐ \| ☐ \| ☐ \| ☐ \| ☐ \| ☐ \| ☐ \| ☐ \| ☐ \| | |
| Time investment | | |
| **Session Delivery**  How much time was spent on the delivery of the session? | | 35-40 minutes |
|  | | |
| \| *(Please tick* ***ONE*** *box on* ***EACH*** *line)* \| Not delivered \| Nothing like description \| Mostly as described \| Exactly as described \| \| --- \| --- \| --- \| --- \| --- \| \| 1. **Welcome and intro** \| ☐ \| ☐ \| ☐ \| ☐ \| \| 1. **Reviewing last week’s goals** \| ☐ \| ☐ \| ☐ \| ☐ \| \| 1. **Setting your SMART goal for next week** \| ☐ \| ☐ \| ☐ \| ☐ \| \| 1. **Gambling-related harms activity** \| ☐ \| ☐ \| ☐ \| ☐ \| \| 1. **Gambling-related harms framework** \| ☐ \| ☐ \| ☐ \| ☐ \| \| 1. **Thinking about gambling harms** \| ☐ \| ☐ \| ☐ \| ☐ \| \| 1. **Additional help** \| ☐ \| ☐ \| ☐ \| ☐ \| \| 1. **Key points recap** \| ☐ \| ☐ \| ☐ \| ☐ \| \|  \| ☐ \| ☐ \| ☐ \| ☐ \| | | |
|  | | |

| **Section** | **Thick Description** *(of participant/facilitator engagement and response to the intervention)* |
| --- | --- |
| **Date, time, location** |  |
| **Facilitators** | . |
| **Participants** |  |
| **1. Welcome Back (5mins)** |  |
| **2. Reviewing last week’s goals (3-4 mins)** |  |
| **3. Setting your SMART goal for next week (2 mins)** |  |
| **4. Gambling-related harms activity** |  |
| **5. Gambling-related harms framework** |  |
| **6. Thinking about gambling harms** |  |
| **7. Recap and additional help (3-5 mins)** | . |
| **8. Next week (0 mins)** | . |

| What worked well? | . |
| --- | --- |
| What did not work well? | . |
| **Specific notes or points of interest or suggestions for improvement.** |  |
